# Molecular responses to therapeutic proteasome inhibitors in multiple myeloma patients are donor-, cell type- and drug-dependent

**DOI:** 10.18632/oncotarget.24882

**Published:** 2018-04-03

**Authors:** Eleni-Dimitra Papanagnou, Evangelos Terpos, Efstathios Kastritis, Issidora S. Papassideri, Ourania E. Tsitsilonis, Meletios A. Dimopoulos, Ioannis P. Trougakos

**Affiliations:** ^1^ Department of Cell Biology and Biophysics, Faculty of Biology, National and Kapodistrian University of Athens, 15784 Athens, Greece; ^2^ Department of Clinical Therapeutics, School of Medicine, National and Kapodistrian University of Athens, 11528 Athens, Greece; ^3^ Department of Animal and Human Physiology, Faculty of Biology, National and Kapodistrian University of Athens, 15784 Athens, Greece

**Keywords:** bortezomib, cancer, carfilzomib, multiple myeloma, proteasome

## Abstract

Proteasome is central to proteostasis network functionality and its over-activation represents a hallmark of advanced tumors; thus, its selective inhibition provides a strategy for the development of novel antitumor therapies. In support, proteasome inhibitors, e.g. Bortezomib or Carfilzomib have demonstrated clinical efficacy against hematological cancers. Herein, we studied proteasome regulation in peripheral blood mononuclear cells and erythrocytes isolated from healthy donors or from Multiple Myeloma patients treated with Bortezomib or Carfilzomib. In healthy donors we found that peripheral blood mononuclear cells express higher, as compared to erythrocytes, basal proteasome activities, as well as that proteasome activities decline during aging. Studies in cells isolated from Multiple Myeloma patients treated with proteasome inhibitors revealed that in most (but, interestingly enough, not all) patients, proteasome activities decline in both cell types during therapy. In peripheral blood mononuclear cells, most proteostatic genes expression patterns showed a positive correlation during therapy indicating that proteostasis network modules likely respond to proteasome inhibition as a functional unit. Finally, the expression levels of antioxidant, chaperone and aggresomes removal/autophagy genes were found to inversely associate with patients’ survival. Our studies will support a more personalized therapeutic approach in hematological malignancies treated with proteasome inhibitors.

## INTRODUCTION

The surveillance of proteome quality is critical for cell survival and is maintained by the modular proteostasis network (PN) [1, 2]. Proteotoxic stress activates the PN in order to either rescue (when feasible), or degrade non repairable polypeptides; a process known as the triage decision of *hold*, *fold*, or *degrade*. Key components of the PN are the protein synthesis module, the Unfolded Protein Response of the Endoplasmic Reticulum (ER) (UPR^ER^) and the molecular chaperones along with the Ubiquitin Proteasome- (UPP) and the Autophagy Lysosome- (ALP) pathways [3, 4]. Part of the PN, are also several transcription factors that mobilize cytoprotective genomic responses upon proteotoxic stress. These (among others) include heat shock factor 1 (HSF1) that activates molecular chaperones [5] and also nuclear factor, erythroid 2 like 2 (NFE2L2, also known as NRF2) that responds to oxidative, electrophilic and/or proteotoxic stress by activating a broad range of phase II and antioxidant enzymes, as well as UPP/ALP genes [6].

UPP is composed of the ubiquitin-conjugating enzymes and the 26S proteasome; it degrades short-lived poly-ubiquitinated normal proteins and non-functional or misfolded polypeptides [4]. The 26S proteasome consists of a catalytic 20S core particle (CP) bound to 19S regulatory particles (RP). The 20S CP is a cylindrical complex being constituted from four stacked heptameric rings; the two outer rings are composed of seven α structural subunits and the two inner rings of seven β catalytic subunits. Eukaryotic proteasomes contain β1, β2 and β5 subunits that bear caspase- (C-L/β1), trypsin- (T-L/β2) and chymotrypsin- (CT-L/β5) like proteolytic activity, respectively [4, 7]. The 19S RP is involved in substrate recognition, deubiquitination, unfolding and translocation into the 20S CP. Most ubiquitinated polypeptides are degraded by the 26S proteasome, whereas non-native (e.g. carbonylated; a type of protein oxidation that can be promoted by Reactive Oxygen Species) or misfolded polypeptides are degraded by the 20S proteasome *via* chaperone-mediated targeting [4, 7]. On the other hand, ALP is an intracellular self-catabolic process that degrades protein aggregates, macromolecules, cytosolic portions and entire organelles *via* lysosomes. In mammalian cells, the most studied forms of autophagy are macroautophagy, microautophagy and chaperone-mediated autophagy [8]. Part of the autophagic processes are also the histone deacetylase 6 (HDAC6) and the sequestosome-1 (SQSTM1, also known as p62) proteins which are, among others, involved in protein aggregates clearance [9].

Deregulation of the PN is associated with several age-related diseases including cancer [1] and it was proposed that proteotoxic stress constitutes a hallmark of cancer [10]. Accordingly, over-activation of PN modules represents a hallmark of advanced tumors, and thus, their inhibition provides a novel strategy for the development of anti-tumor therapies. In line with this notion, proteasome inhibitors (PIs), e.g. Bortezomib (BTZ) and Carfilzomib (CFZ), have demonstrated clinical efficacy in the treatment of Multiple Myeloma (MM) and Mantle Cell Lymphoma (MCL) and are under evaluation for the treatment of other malignancies [11, 12]. Both BTZ and CFZ were designed to target the rate limiting for protein breakdown CT-L proteasomal activity [13, 14], where BTZ binds in a slowly reversible manner and CFZ binds irreversibly [15]. Nevertheless, and despite the introduction of new therapies (e.g. specific PIs) and the noted increase in overall survival rates, MM is in general incurable. Thus, considering that treated patients usually become refractory to therapeutic PIs, which also exert several adverse effects [16, 17], the necessity for in depth understanding of the triggered molecular responses in tumor and/or normal cells upon proteasome inhibition is urgent.

By using *Drosophila* flies as a model organism to study the *in vivo* molecular consequences of partial proteasome inhibition in higher metazoans tissues, we recently reported that proteasome regulation is tissue- and age-dependent. We also found that administration of PIs in young flies resulted in loss of proteostasis, NRF2-dependent upregulation of proteasomal genes and eventually collapse of proteostasis. In terms of pathophysiological effects, administration of PIs in flies caused premature aging, as well as developmental and neuromusculatory defects [18, 19].

Herein, we studied proteasome functionality in human healthy donors and in MM patients treated with therapeutic BTZ or CFZ. In healthy donors we noted that peripheral blood mononuclear cells (PBMCs) express higher proteasomal activities as compared to anucleated red blood cells (RBCs), as well as that proteasome activities decline during aging in both cell types. Studies in cells isolated from MM patients treated with PIs revealed donor-, cell type-, and drug-specific readouts. Notably, the expression of antioxidant, chaperone and aggresomes removal/autophagy genes in PBMCs inversely associated with patients’ survival rates.

## RESULTS

### Basal proteasome activities in PBMCs and RBCs from healthy donors are characterized by significant variability and decline during aging

**Figure 1:**
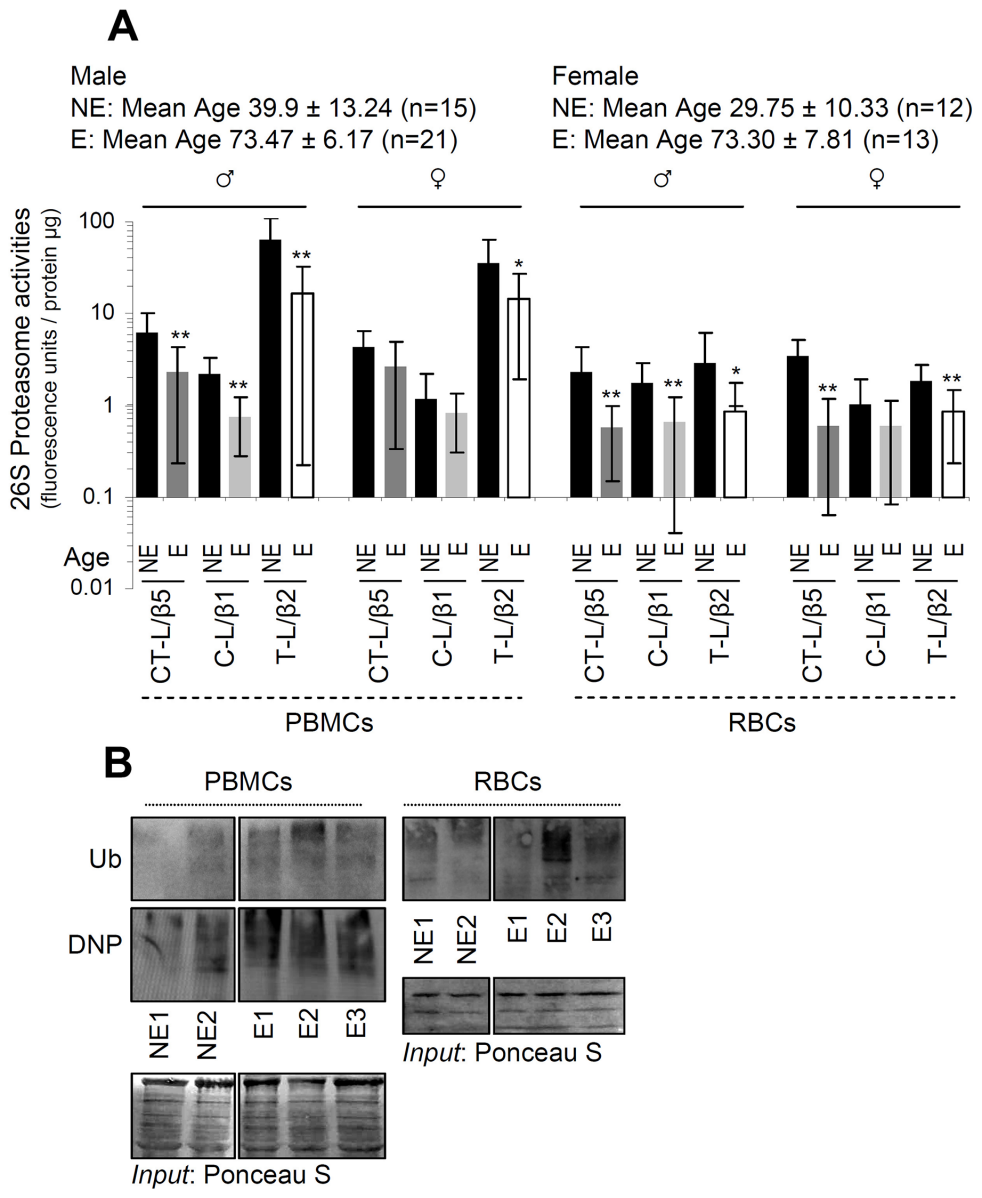
Basal proteasome activities in both PBMCs and RBCs decline during aging **(A)** Relative activity of the CT-L (β5), C-L (β1) and T-L (β2) proteasome peptidases activities in PBMCs and RBCs from healthy nonelderly (NE) or elderly (E) donors; mean age and number of donors per group are indicated. **(B)** Representative immunoblotting analyses of ubiquitin (Ub) or carbonylation (DNP) in lysates from PBMCs and RBCs isolated from nonelderly or elderly healthy donors. Staining of nitrocellulose membranes with Poncaeu S was used as reference for total protein input. Ages of donors in (B): NE1: 24, NE2: 26, E1: 80, E2: 84, E3: 89. Bars, ± SD. ^*^P < 0.05; ^**^P < 0.01.

**Figure 2:**
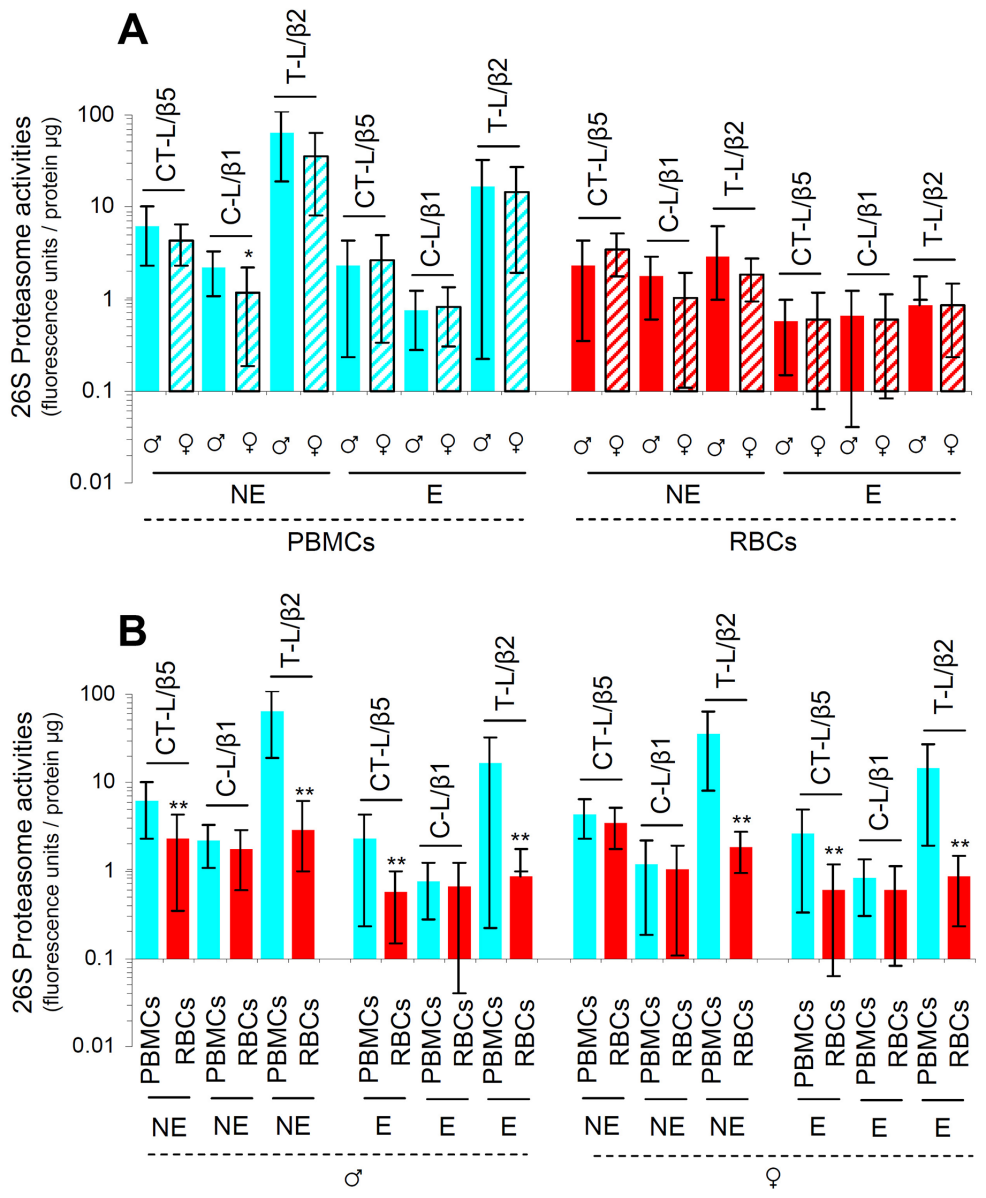
PBMCs express higher basal proteasome activities as compared to RBCs in both male and female donors Proteasome activities in PBMCs and RBCs isolated from nonelderly (NE) or elderly (E) (see Figure 1) healthy donors. **(A)** Males’ PBMCs showed higher C-L enzymatic activities as compared to PBMCs from females of similar age; other noted differences did not reach statistical significance. **(B)** PBMCs express higher basal proteasome activities as compared to anucleated RBCs. Top of each bar refers to the mean. Bars, ± SD. ^*^P < 0.05; ^**^P < 0.01.

As we observed that one cycle of deep freeze-thawing in isolated PBMCs or RBCs resulted in diminished proteasome activities (Supplementary Figure 1), all downstream assays were performed in freshly isolated samples. We found that proteasomal activities decreased (in a sex-independent manner) during aging in both PBMCs and RBCs (Figure 1A). In support, immunoblotting analyses in PBMCs and RBCs from nonelderly and elderly healthy donors revealed increased proteome ubiquitination and carbonylation during aging (Figure 1B) indicating UPP dysfunction. Additionally, in spite of a tendency for higher basal proteasomal activities in cells isolated from nonelderly males *vs*. females these differences were in most cases not statistically significant (Figure 2A). Interestingly enough, data reorganization revealed that PBMCs express higher, as compared to RBCs, basal proteasome activities in both male and female donors of similar age (Figure 2B). Thus, despite donor specific variability the basal proteasomal activities in peripheral blood cells are cell type- and age-dependent.

### Treatment of MM patients with therapeutic doses of BTZ or CFZ results in donor-specific readouts

**Figure 3:**
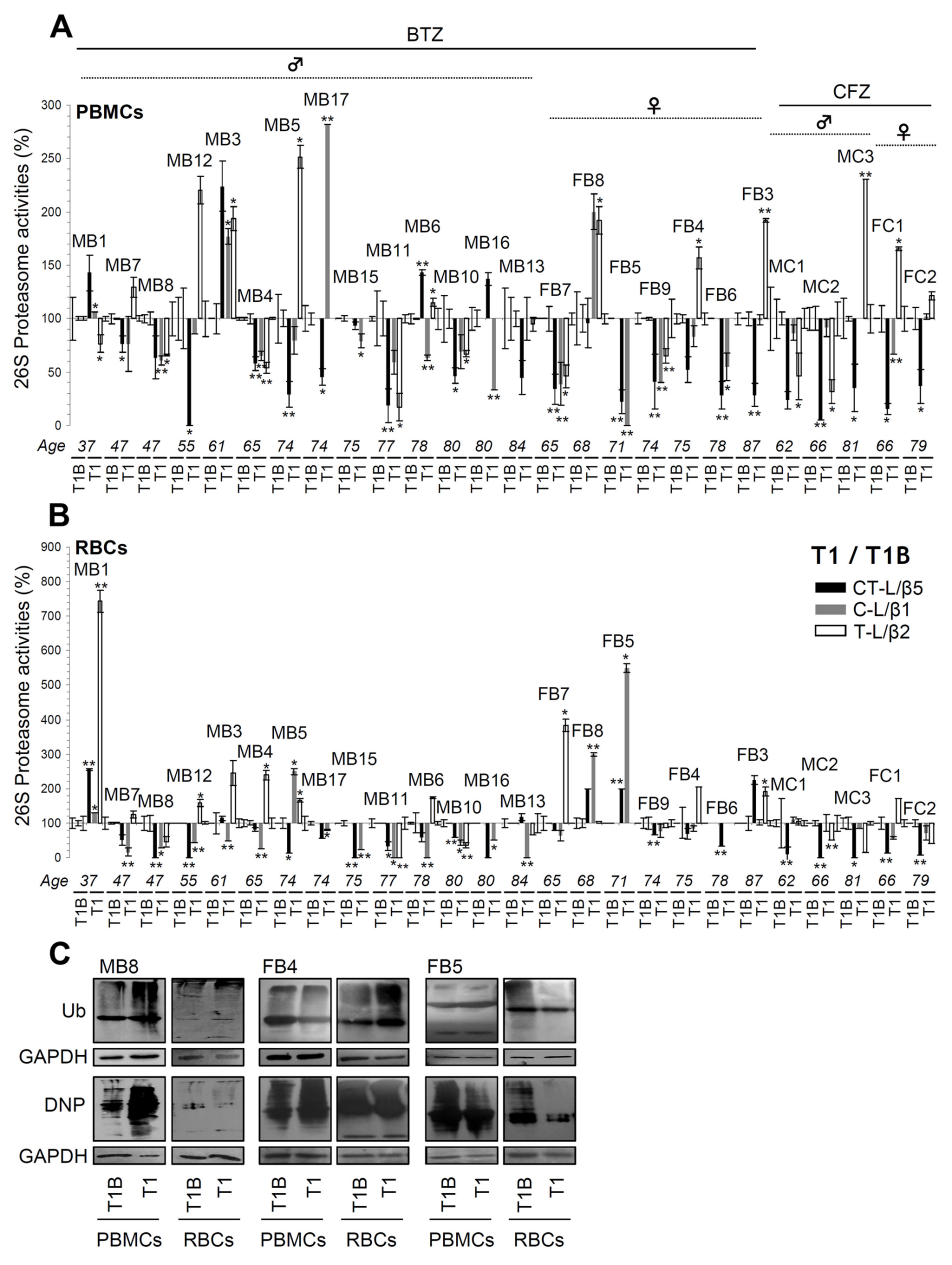
Proteasome activities in PBMCs and RBCs twenty four hrs post-treatment (time point T1) of MM patients with BTZ or CFZ Relative (%) CT-L, C-L and T-L proteasome activities in PBMCs **(A)** and RBCs **(B)** isolated from MM patients (n=26) after receiving therapeutic doses of BTZ or CFZ for 24 hrs (time point T1; see Supplementary Figure 2). Percent of proteasome activities as compared to basal values in either PBMCs (A) or RBCs (B) isolated before treatment initiation (T1B) are shown. **(C)** Representative immunoblotting analyses of PBMCs and RBCs samples isolated from MM patients at T1; protein samples were probed with antibodies against ubiquitin (Ub) or protein carbonylation (DNP). GAPDH probing was used as reference for total protein input. MB: Male BTZ; FB: Female BTZ; MC: Male CFZ; FC: Female CFZ. Bars, ± SD. ^*^P < 0.05; ^**^P < 0.01.

To examine the molecular effects of PIs on human peripheral blood cells, we then assayed proteasome activities at different time points (Supplementary Figure 2) during patients’ treatment. We observed that in most (but, interestingly enough, *not all*) patients proteasome activities were suppressed in both PBMCs (Figure 3A) and RBCs (Figure 3B) twenty four hours (T1) post-drug administration. In subsequent time points (i.e. T3B, T3 and C2B; see, Supplementary Figure 2), we found that in most MM patients proteasome activities remained low (in both cell types) after administration of either BTZ or CFZ (Supplementary Figures 3A, 3B, 4, 6). Worth mentioning is that, as expected, RBCs that lack genomic responses were particularly sensitive to the PIs. Also, CFZ showed increased (*vs*. BTZ) selectivity against the CT-L proteasome activity (Figure 3A, 3B; and Supplementary Figures 3A, 3B, 4, 6); this property likely relates to CFZ reduced toxicity and side-effects in the clinic [11].

An unexpected finding was that in some patients, proteasomal activities of either PBMCs or RBCs increased despite administration of PIs. This response appeared either at T1 (e.g. PBMCs from patients MB1, MB3 and MB6 or RBCs from patients MB1 and FB8) or in later therapeutic cycles (e.g. PBMCs from patients MB12, MB3 and MB13 or RBCs from patients MB8, MB12, MB11, MB13 and FB7) (Supplementary Figures 3A, 3B, 4, 6). Notably, the BTZ or CFZ treatment-mediated effects on peripheral blood cells proteasomal activities showed minimal correlation with the levels of isolated PBMCs or RBCs proteome ubiquitination and/or carbonylation (e.g. patients MB8, FB4 and FB5) (Figure 3C) (see also, Supplementary Figures 3C, 5) indicating that other proteostatic modules are likely activated (in a patient-specific manner) in order to alleviate proteotoxic stress and proteome instability.

**Figure 4:**
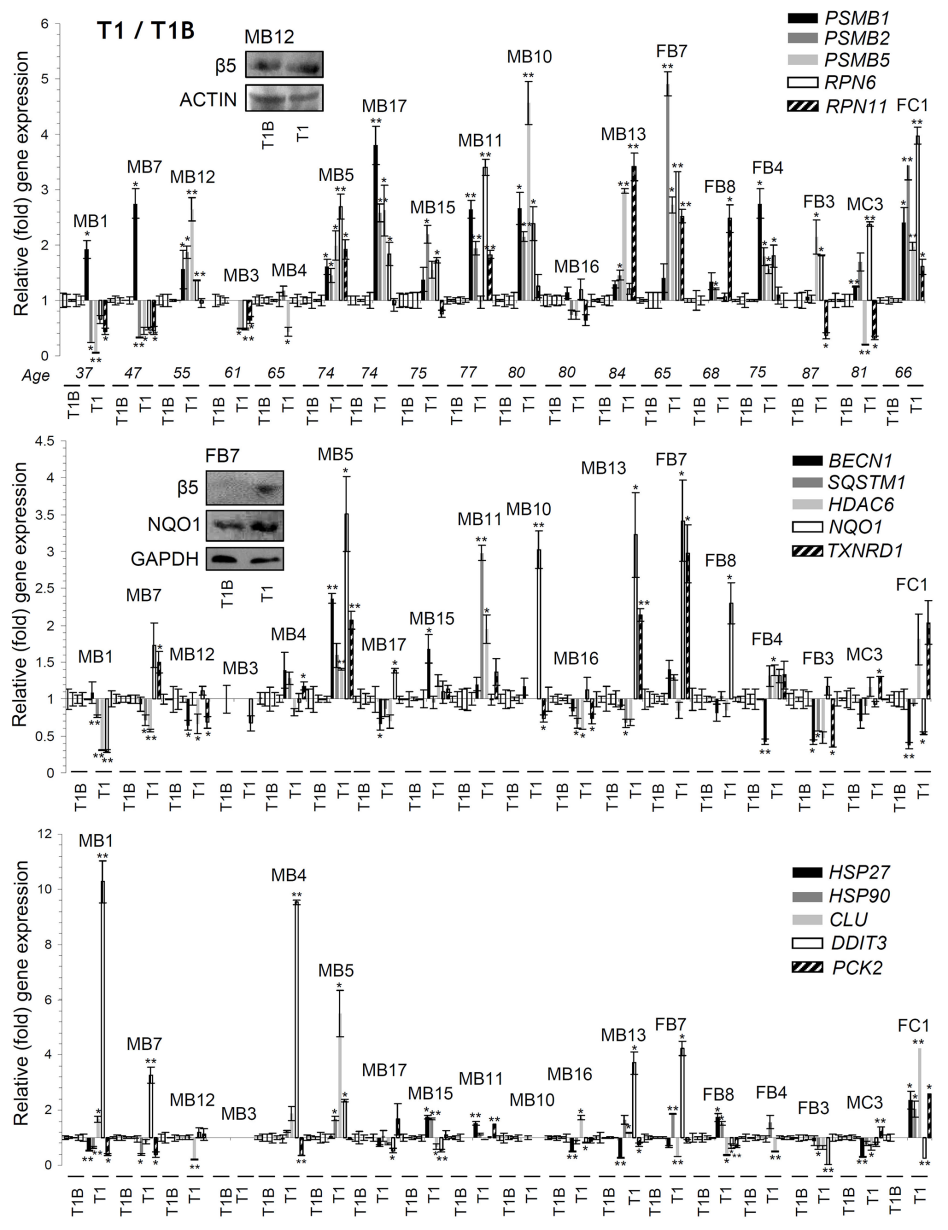
Differential genomic responses in PBMCs twenty four hrs post-treatment (T1) of MM patients with BTZ or CFZ Relative expression levels of the *PSMB1*, *PSMB2*, *PSMB5*, *RPN6*, *RPN11* (proteasome), *BECN1*, *SQSTM1*, *HDAC6* (autophagic/aggresomes removal), *NQO1*, *TXNRD1* (antioxidant), *HSP27*, *HSP90*, *CLU* (chaperone), *DDIT3* (UPR^ER^) and *PCK2* (metabolic) genes in PBMCs isolated from MM patients 24 hrs post-treatment with therapeutic doses of BTZ or CFZ (T1; see Supplementary Figure 2). Inserts show immunoblotting analyses of PBMCs samples isolated from indicated MM patients at T1; protein samples were probed with antibodies against proteasomal β5 or NQO1. Normalization of gene expression was *vs*. basal gene expression values (set to 1) found in samples isolated before treatment initiation (T1B). ACTIN or GAPDH probing and *GAPDH* gene expression were used as reference for total protein and total RNA input, respectively. MB: Male BTZ; FB: Female BTZ; MC: Male CFZ; FC: Female CFZ. Bars, ± SD. ^*^P < 0.05; ^**^P < 0.01.

We then investigated in PBMCs the effects of the administrated PIs on the expression of proteostatic genes. As shown in Figure 4 (and in Supplementary Figures 7, 9, 10) BTZ or CFZ treatment triggered in most (but again, *not all*) patients the upregulation of proteasome genes (*RPN6, RPN11, PSMB1, PSMB2, PSMB5*) expression, indicating that the feedback regulatory circuit which functions to restore normal proteasome activities in flies [19] is also operational in humans. Additional gene (or protein; see inserts in Figure 4) expression analyses in PBMCs showed in several patients the upregulation of genes involved in autophagy and aggresomes removal (*BECN1*, *SQSTM1*, *HDAC6, CTSL, CTSD*); in antioxidant-responses (*NQO1*, *TXNRD1*), as well as in chaperon [*HSP27* (also known as *HSPB1*), *HSP90* (also known as *HSP90AA1*), *CLU*], unfolded protein responses (*DDIT3,* also known as *CHOP*) and metabolic (*PCK2*, also known as *PEPCK*) pathways (Figure 4, Supplementary Figures 7-10).

In relation to gene expression patterns in PBMCs we found at time point T1 a positive correlation of most genes assayed (Supplementary Tables 1, 2) indicating that likely PN modules are regulated upon proteasome inhibition as a highly integrated functional unit. This positive correlation mainly referred to proteasomal genes and to autophagic/aggresomes removal *BECN1*, *SQSTM1* and *HDAC6* genes. Also, during therapy the *SQSTM1* and *HDAC6* genes expression correlated positively with the expression levels of molecular chaperones (e.g. *HSP27* and *HSP90*) genes. Interestingly, the BTZ or CFZ treatment-mediated gene expression patterns showed negative correlation with proteasome peptidases activities, suggesting the existence of a regulatory network that modulates proteasomal genes expression in order to achieve an evolution-set “optimum” of physiological proteasome peptidase activities. Taken together these data indicate that treatment of MM patients with therapeutic doses of BTZ or CFZ led to donor-specific responses in peripheral blood cells.

### Regulation of proteostatic modules at peripheral blood cells showed significant correlations with survival rates of BTZ-or CFZ-treated MM patients

We further sought to identify potential associations of the aforementioned proteostatic modules (e.g. proteasomal activities or gene expression) regulation with clinical outcomes, i.e. depth of response according to the IMWG criteria, progression free patient survival (PFS) and overall patient survival (OS) (see Supplementary Tables 1, 2). We found that basal proteasome activities in PBMCs correlate positively with each other and with activities in RBCs indicating that proteasome functionality is likely regulated across tissues at a systemic level. In support, we observed a positive correlation in proteasomal activities of PBMCs and RBCs after drug treatment likely suggesting common responses in the bone marrow rates of haemopoiesis. Furthermore, the *TXNRD1* (antioxidant), *HSP27* (chaperone) and *HDAC6* (autophagy/aggresomes removal) genes expression (at various time points during therapy) were inversely associated with PFS; also, the *SQSTM1* (autophagy/aggresomes removal) gene expression was inversely associated with both PFS and OS. In conclusion, upregulation of proteostatic genes during therapeutic treatment of MM patients with PIs likely correlates with less favorable outcomes after PI-based therapy.

## DISCUSSION

Proper UPP functionality is central to proteostasis maintenance since it represents a major regulatory hub for cellular survival in both physiological and cancer cells. Hence it is not surprising that the usage of PIs has revolutionized cancer therapy [20]. Specifically, PIs have demonstrated clinical efficacy in the treatment of hematological cancers and are under evaluation for other malignancies [11]. Considering however that the primary responses to therapeutic PIs in treated MM patients are not well understood; and the necessity for a more *personalized therapeutic approach*, we analyzed the functionality of proteostatic modules in PBMCs and RBCs isolated from either healthy donors or from MM patients treated with PIs. In healthy donors we noted that proteasome peptidase activities in both cell types decreased during aging. In support, previous studies have shown that proteasome activity declines during mammalian cells’ senescence [21]; in cells derived from elderly donors [22], as well as during aging in various rat and mouse tissues including adipose tissue, spinal cord, heart, brain, liver and muscle [4]. Likely, age-related reduced proteasome activity relates to either proteasome clogging due to increased formation of protein aggregates [23, 24] or to decreased proteasome subunits expression and/or assembled proteasomes [18]. Interestingly, we found that PBMCs express higher proteasome activities, as compared to anucleated highly differentiated RBCs. It can thus be assumed that the therapeutic window observed in MM by using PIs could also relate to the fact that highly proliferating (or less differentiated) tumor cells are “addicted” to increased UPP activities (due also to enhanced protein synthesis and elevated proteotoxic stress) and are therefore differentially sensitive to proteasome inhibition.

As anticipated, in most MM patients treatment with PIs resulted in reduced proteasomal activities in both PBMCs and RBCs. In some cases, proteasome inhibition was achieved in later therapeutic cycles while (mostly in PBMCs) proteasome activities rebound during the periods of no drug administration. Also, as in flies’ tissues [19], proteasome inhibition led in some patients’ PBMCs to the upregulation of proteostatic genes. Thus, the feedback regulatory circuit which functions to restore proteome stability upon proteasome dysfunction is highly conserved in higher metazoans. Proteostatic modules showed a positive correlation 24 hours (T1) post-drug administration indicating that likely they are regulated as a highly wired integrated functional unit. This was particularly evident by the identified positive correlation of proteasomal genes with the autophagic *SQSTM1*, *HDAC6* and *BECN1* genes, as well as by the positive correlation of the latter with the expression levels of chaperone (e.g. *HSP27* and *HSP90*) genes. Therefore, proteasome dysfunction mobilizes the induction of most of the main PN modules. In support, PIs have been previously implicated in the induction of HSP70 [25, 26] and in the activation of prosurvival autophagy in mammalian cells *via* (among others) UPR induction [27]. The activation of additional PN modules (e.g. ALP) upon UPP inhibition is also supported by our finding of minimal correlation between the levels of proteome ubiquitination/carbonylation in peripheral blood cells and the level of proteasome activity downregulation. These findings broaden the potential means for increasing the therapeutic window against malignancies, e.g. by also using inhibitors of ALP. Consistently, a recent study revealed that a combination therapy of CFZ and chloroquine (an ALP inhibitor) was highly effective in the treatment of MM in a mouse xenograft model [28].

In line with these notions, we noted a negative correlation between the expression of the *TXNRD1*, *HSP27*, *HDAC6* and *SQSTM1* genes and the patients’ PFS and/or OS (Table 1, Supplementary Table 3) indicating that likely the upregulation of these genes during therapy represents a prognostic marker for poor outcome. In support, blockade of HSP27 overcomes BTZ resistance in lymphoma cells [29] while in a mouse myeloma model, resistance-related gene signatures were enriched for expression of *nrf2* [30]. Also, increased expression of antioxidant genes has been associated with resistance to treatment with BTZ in patients with leukemic MCL [31]. Furthermore, POMP (a chaperone that participates in the assembly of active proteasome particles from inactive precursor subunits) is an NRF2 transcriptional target and a mediator of BTZ resistance [32].

**Table 1:** Parameters found to show significant correlation with IMWG-related criteria for depth of response to therapy, PFS and OS in both Pearson’s (parametrical) and Spearman’s (non parametrical) correlation analyses (see also Supplementary Tables 1-3)

Pearson									
	T-L_BASAL_RBCs	*HSP27* _1	*HDAC6* _3	*TXNRD1* _3	*RPN11* _C2B	*SQSTM1* _C2B	*NQO1* _C2B	IMWG	PFS
**IMWG**							-0.83^*^		
**PFS**	-0.47^*^	-0.519^*^	-0.707^*^	-0.761^*^	0.989^**^	-0.917^*^		-0.801^**^	
**OS**						-0.882^*^		-0.695^**^	0.767^**^
**Spearman’s**									
	**T-L_BASAL_RBCs**	***HSP27* _1**	***HDAC6* _3**	***TXNRD1* _3**	***RPN11* _C2B**	***SQSTM1* _C2B**	***NQO1* _C2B**	**IMWG**	**PFS**
**IMWG**							-0.828^*^		
**PFS**	-0.448^*^	-0.527^*^	-0.745^*^	-0.848^**^	0.949^**^	-0.959^*^		-0.815^**^	
**OS**						-0.891^*^		-0.64^**^	0.767^**^

T-L_BASAL_RBCs, Basal T-L activity in RBCs before administration of PIs; *HSP27*_1, gene expression at T1; *HDAC6*_3, gene expression at T3; *TXNRD1*_3, gene expression at T3; *RPN11*_C2B, gene expression at C2B; *SQSTM1*_C2B, gene expression at C2B; *NQO1*_C2B, gene expression at C2B. IMWG (International myeloma working group criteria of response to therapy), PFS (Period of progression free patient survival), OS (Period of overall patient survival). ^*^P<0.05; ^**^P<0.01.

As the impact of *HDAC6* and *SQSTM1* genes expression on MM remain largely unknown, our study adds novel data for their potential prognostic value in relation to MM patients' outcome. The major substrate of HDAC6 is α-tubulin and this protein had been involved in the ALP-mediated removal of aggresomes [33]. On the other hand, SQSTM1 is an ubiquitin-binding autophagic adaptor being involved in the removal of protein aggregates and damaged organelles (e.g. mitochondria) *via* ALP [34]; reportedly, SQSTM1 interacts with HDAC6 and downregulates its deacetylase activity [35]. HDAC6 is involved in the activation of HSF1 (a transcriptional activator of the *HSP27* gene) [36] and SQSTM1 has been implicated in activating NRF2 [37, 38] and thus cellular antioxidant responses. Consequently, a selective HDAC6 inhibitor (ACY-241) enhanced pomalidomide anti-tumor response in MM [39] and ricolinostat (ACY-1215) is in test in combination with BTZ and dexamethasone for relapsed or refractory MM [40]. Furthermore, and in line with our findings, it was recently reported that deregulation of histone deacetylases is an indicator of poor prognosis in MM [41]. Similarly, it has been reported that activation of the SQSTM1-NRF2 pathway mediates PIs resistance in MM cells *via* redox, metabolic and translational reprogramming [42]. Also, the SQSTM1-KLF4-associated prosurvival autophagy contributes to CFZ resistance in MM models [43], while blocking SQSTM1 suppresses myeloma growth and osteoclast formation *in vitro* and induces bone formation in myeloma-bearing bones *in vivo* [44].

Of particular interest is our finding that despite treatment with PIs, the PBMCs, and even more interestingly RBCs, of several patients expressed elevated proteasomal activities during therapy. In the case of PBMCs these responses may relate to gene expression alterations that trigger the overexpression of UPP components in order to surpass the inhibitory effects of the PIs. In most cases induction of proteasome genes is an early response that results in downstream enhanced production of assembled proteasomes and thus increased enzymatic activities/μg of total cellular protein. In support, overexpression of the proteasomal subunit β5 (or other subunits, such as β2 and β1) has been reported to act as a possible mechanism of resistance to BTZ [45-47], while in line with the notion of a counteracting transcriptional mechanism that neutralizes the PIs’ effects, no *PSMB5* mutations were identified in BTZ-resistant patients [12, 48-50]. In the case of the anucleated RBCs higher proteasome activities may relate to increased clearance of damaged (e.g. prematurely senescent) RBCs *via* erythrophagocytosis that may lead to bone marrow-mediated increased erythropoiesis or proteasome load in newly produced RBCs. In support, erythropoiesis was enhanced in a mouse model after lactic acid-mediated oxidative stress (which is also a side-effect of PIs treatment [19]) in the bone marrow [51]. Erythropoiesis also increases after hemolysis, hemorrhage [52] or during hypoxia [53] indicating the activation of systemic responses upon loss of peripheral blood homeodynamics and/or bone marrow functionality. In other words, during therapy apart from “topical” cellular responses, the systemic (e.g. bone marrow-mediated) counteracting mechanisms should be also considered and studied in more detail.

Taken together, our observations in MM patients largely recapitulate the findings in the fly model [19] indicating that *Drosophila* represents a unique *in vivo* experimental platform for studying proteostatic modules during normal aging and in diseases in order to distinguish between correlation and causation; as well as, for screening the efficacy and/or safety of PIs or other drugs [19, 54, 55]. Given that proteasomal activities decline in physiological tissues during aging, it is anticipated that the toxicity of PIs will be significantly increased in elderly patients, as it has been clinically observed. Our data also raise the necessity of more detailed studies in larger cohorts and of measuring (or, even better, adjusting) the levels of achieved reduction in proteasomal activities after administration of therapeutic PIs (preferentially by using noninvasive methods in peripheral blood cells) and not simply delivering the drugs in doses of mg/m^2^. Finally, the characterization of the differential dependence of mitotic and post-mitotic tissues in the distinct PN modules (e.g. UPP *vs*. ALP), along with the understanding of the mechanistic details of bone marrow responses to PIs and also whether these responses are mirrored in peripheral blood cells, remain a future challenge in order to monitor therapy evolvement; to understand mechanisms of innate and acquired resistance and to avoid clinical trial failures.

## MATERIALS AND METHODS

### Participating subjects

Informed consent was obtained from all healthy individual participants that were included in the study; mean ages per sex, and numbers of donors are shown in Figure 1. Considering ethics committee approval and informed consent, all 26 patients that were included in this study were diagnosed with symptomatic MM and were previously untreated. Patients were treated with the indicated PI (either CFZ or BTZ) at the Department of Clinical Therapeutics, “Alexandra” Hospital (Athens, Greece). The mean age of male patients was 67 years ± 13.95 (range 37-84) whereas the mean age of female patients was 74 years ± 6.67 (range 65-87). Healthy subjects or MM patients only donated blood.

### Blood and PBMCs, RBCs isolation

PBMCs and RBCs were collected by using Biocoll [density 1.077 g/ml (Biochrom)] from freshly collected heparinized blood, that was transferred into a 15 mL tube and was then diluted (1:1 dilution) with phosphate-buffered saline (PBS). The diluted whole blood was then carefully layered over the separation medium (1/2 x the volume of the sample); the two phases were kept separated before the centrifugation. Tubes were then centrifuged at 400 xg for 30 min at 20°C (acceleration 9, braking rate 0). PBMCs were collected after aspiration of the plasma and platelets layer. For RBCs isolation, the upper layer of polymorphonuclear leukocytes and a part of the RBCs phase were also aspirated leaving RBCs sediment undisturbed; the highly enriched fraction of RBCs was then collected. Both cell types were washed with PBS and were then processed for downstream assays.

### RNA extraction and quantitative real-time PCR (Q-RT-PCR) analyses

Total RNA was extracted from PBMCs using the NucleoSpin^®^ RNA mini kit (Macherey Nagel) and quantified with a BioSpec-nano spectrophotometer (Shimadzu Inc.). RNA (100 ng) was converted to cDNA with the Maxima First Strand cDNA Synthesis Kit for RT-qPCR (Thermo Scientific). Real-time PCR (in duplicates) was performed using Maxima SYBR Green/ROX qPCR Master Mix (Thermo Scientific) and the PikoReal 96 Real-Time PCR System. All oligonucleotides used in this work were designed using the primer-BLAST tool (http://www.ncbi.nlm.nih.gov/tools/primer-blast/). The RT-PCR gene-specific primers are reported in Supplementary Materials and Methods.

### Measurement of proteasome peptidase activities

For measuring proteasome peptidases activity, cells were lysed on ice in a buffer suitable for isolation of intact 26S proteasomes; protein content was adjusted with Bradford and cleared supernatants were immediately used (after the addition of the fluorogenic substrates) to determine the three proteasome proteolytic activities as described [18]. Emitted fluorescence was recorded at a VersaFluor Fluorometer System (Bio-Rad laboratories, Hercules, CA, USA) at excitation and emission wave lengths of 350 and 440 nm, respectively.

### Preparation of cell protein extracts, immunoblot analysis and detection of protein carbonyl groups

Isolated cells were lysed on ice in NP-40 lysis buffer containing protease inhibitors and were analyzed by SDS-PAGE and immunoblotting as described previously [18, 56]. Primary and secondary antibodies were applied for 1 h at RT; blots were developed by an enhanced chemiluminescence reagent kit (Clarity™ Western ECL Blotting Substrate, Bio-Rad).

Protein carbonylation refers to a protein oxidation process that forms reactive ketones or aldehydes that can react with 2,4-dinitrophenylhydrazine (DNPH) to form hydrazine [57]. For the detection of protein carbonyl groups the OxyBlot protein oxidation detection kit (Millipore, Billerica, MA; #s7150) was used as per manufacturer’s instructions.

### Antibodies used

Primary antibodies against ACTIN (#1615), β5 proteasomal subunit (#55009), NQO1 (#16464), Ubiquitin (#8017) and the HRP-conjugated secondary antibodies were purchased from Santa Cruz Biotechnology; the anti-GAPDH (G9545) antibody was from Sigma-Aldrich.

### Clinical data and statistical analysis

Data were collected from the medical files of the enrolled patients. Disease-related measures (depth of response, PFS, OS) were recorded according to IMWG (International Myeloma Working Group) criteria [58]. PFS and OS were measured from the day of treatment initiation to disease progression or death from any cause. Assays were performed at least in duplicates. Statistical significance was evaluated by using Student’s *T* test (2-tailed) or by using ANOVA single factor (Alpha = 0.05). Error bars denote standard deviation (S.D.); significance at P<0.05 or P<0.01 is indicated on the graphs by one or two asterisks, respectively. For S.D. calculation in Excel the STDEV function was used and the output is recorded on the graphs. S.D. points at T1B refer to variation of the independent measurements; the mean of which was set to 100%. Pearson’s (r) and Spearman’s Rank (rho) correlation coefficient tests were employed to examine relationships between various parameters and clinical data. Analyses were performed using either Microsoft Excel or SPSS 23 software (IBM SPSS statistics for Windows, Version 23.0, IBM Corp, Armonk, NY, USA).

### Ethics approval and consent to participate

Informed consent was obtained from all individual participants included in the study, which was conducted in accordance with the ethical standards of the institutional and/or national research committee and with the 1964 Helsinki Declaration and its later amendments or comparable ethical standards. An approval has been obtained from the Institutional Review Board/Scientific committee of “Alexandra” Hospital (Athens, Greece) for the collection of blood samples.

### Availability of data and material

Materials used and the datasets generated and/or analyzed during the current study are available from the corresponding authors on reasonable request.

## SUPPLEMENTARY MATERIALS FIGURES AND TABLES





## Abbreviations

ALP: Autophagy lysosome pathway
C-L/β1: Caspase-like proteasomal activity
CT-L/β5: Chymotrypsin-like proteasomal activity
HDAC6: Histone deacetylase 6
HSF1: Heat shock transcription factor-1
HSP: Heat shock protein
IMWG: International myeloma working group
MCL: Mantle cell lymphoma
NFE2L2: Nuclear factor, erythroid 2 like 2
OS: Overall survival
SQSTM1: sequestosome-1
PCK2: Phosphoenolpyruvate carboxykinase 2, mitochondrial
PDR: Proteome damage response
PFS: Progression free survival
PN: Proteostasis network
T-L/β2: Trypsin-like proteasomal activity
Ub: Ubiquitin
UPP: Ubiquitin proteasome pathway
UPR: Unfolded protein response
