# Supplementary material for: Molecular responses to therapeutic proteasome inhibitors in multiple myeloma patients are donor-, cell type- and drug-dependent
Source: Oncotarget. 2018 Apr 3;9(25):17797–809. doi: 10.18632/oncotarget.24882 (PMC5915155; doi:10.18632/oncotarget.24882)
Supplement: Supplementary file 1 [file oncotarget-09-17797-s001.pdf]

Molecular responses to therapeutic proteasome inhibitors in multiple myeloma patients are donor-, cell type- and drug-dependent

SUPPLEMENTARY MATERIALS

Q-RT-PCR gene specific primers

The RT-PCR-gene specific primers used were the following: (5'→3') (*F for forward primer, R for reverse primer). PSMB1*-F: GGA-TGC-AGC-GGT-TTT-CAT-GG, *PSMB1*-R: AAT-TGC-CCC-CGT-AGT-CAT-GG; *PSMB2*-F: CTG-CTC-CGC-CCT-CCA-TTA-AC, *PSMB2*-R: GCC-AAG-CAT-GGA-GTA-GAA-CG; *PSMB5*-F: TCA-AGT-TCC-GCC-ATG-GAG-TC, *PSMB*5-R: CTT-CTT-CAC-CGT-CTG-GGA-GG; *RPN6*-F: TCA-AAC-TCT-CCA-AGG-CCG-AC, *RPN6*-R: CTC-CCC-CTG-GTC-CAA-AAT-CC; *RPN11*-F: ACG-GAA-GCC-GAA-GCA-AAC-TA, *RPN11*-R: GCA-AAC-CGG-CGA-TGA-ATC-AG; *BECN1*-F: AAC-CAG-ATG-CGT-TAT-GCC-CA, *BECN1*-R: TCC-ATT-CCA-CGG-GAA-CAC-TG; *SQSTM1*-F: CAT-CGG-AGG-ATC-CGA-GTG-TG, *SQSTM1*-R: TTC-TTT-TCC-CTC-CGT-GCT-CC; *HDAC6*-F: GAC-CAT-CCA-AGT-CCA-TCG-CA, *HDAC6*-R: ACC-TAG-GTT-TGG-CTG-GTT-GG; *NQO1*-F: AGC-AGA-CGC-CCG-AAT-TCA-AA, *NQO1*-R: AGA-GGC-TGC-TTG-GAG-CAA-AA; *CT SL*-F: ACA-GGG-AAG-GGA-AAC-ACA-GC, *CTSL*-R: TTC-ACA-GGA-GTC-ACG-TAG-CC; *CTSD*-F: ACC-TTC-ATC-GCA-GCC-AAG-TT, *CTSD*-R: AGC-ACG-TTG-TTG-ACG-GAG-AT; *TXNRD1*-F: TTG-GAG-TGC-GCT-GGA-TTT-CT, *TXNRD1*-R: TTT-GTT-GGC-CAT-GTC-CTG-GT; *HSP27*-F: CCA-CCC-AAG-TTT-CCT-CCT-C, *HSP27*-R: GAC-TGG-GAT-GGT-GAT-CTC-GT; *HSP90*-F: GGC-AGA-GGC-TGA-TAA-GAA-CG, *HS P90*-R: CTG-GGG-ATC-TTC-CAG-ACT-GA; *CLU*-F: AAA-CGA-AGA-GCG-CAA-GAC-AC, *CLU*-R: TGT-TTC-AGG-CAG-GGC-TTA-CA; *DDIT3*-F: CGA-CAG-AGC-CAA-AAT-CAG-AGC, *DDIT3*-R: TTC-AGG-TGT-GGT-GAT-GTA-TGA-AGA; *PCK2*-F: GAA-GTG-CTT-TGC-CCT-ACG-C, *PCK2*-R: CCC-CCA-CAC-ACT-CCA-CTT-TC. The *GAPDH* gene (*GAPDH*-F: CCA-CAT-CGC-TCA-GAC-ACC-AT, *GAPDH*-R: CCA-TGG-GTG-GAA-TCA-TAT-TGG-AAC) was used as a normalizer.

SUPPLEMENTARY FIGURES AND TABLES

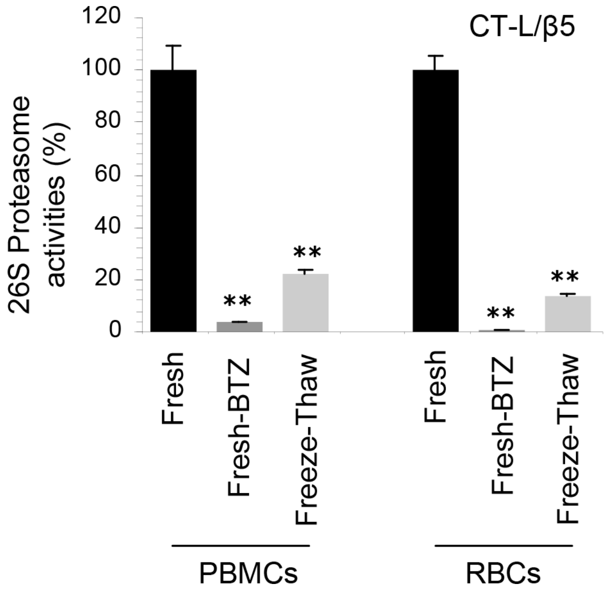

Supplementary Figure 1: One cycle of *deep freeze-thawing* in isolated PBMCs or RBCs results in significant loss of proteasome peptidases activities. Relative (%) proteasome activities in fresh *vs.* deep frozen (-80°C) - thawed (1 cycle) isolated PBMCs or RBCs. Bortezomib (Fresh-BTZ) addition in cell lysates minimized readout, verifying the specificity of the assay; shown data refer to the CT-L (β5) peptidase activity. Bars, ± SD. **P < 0.01.

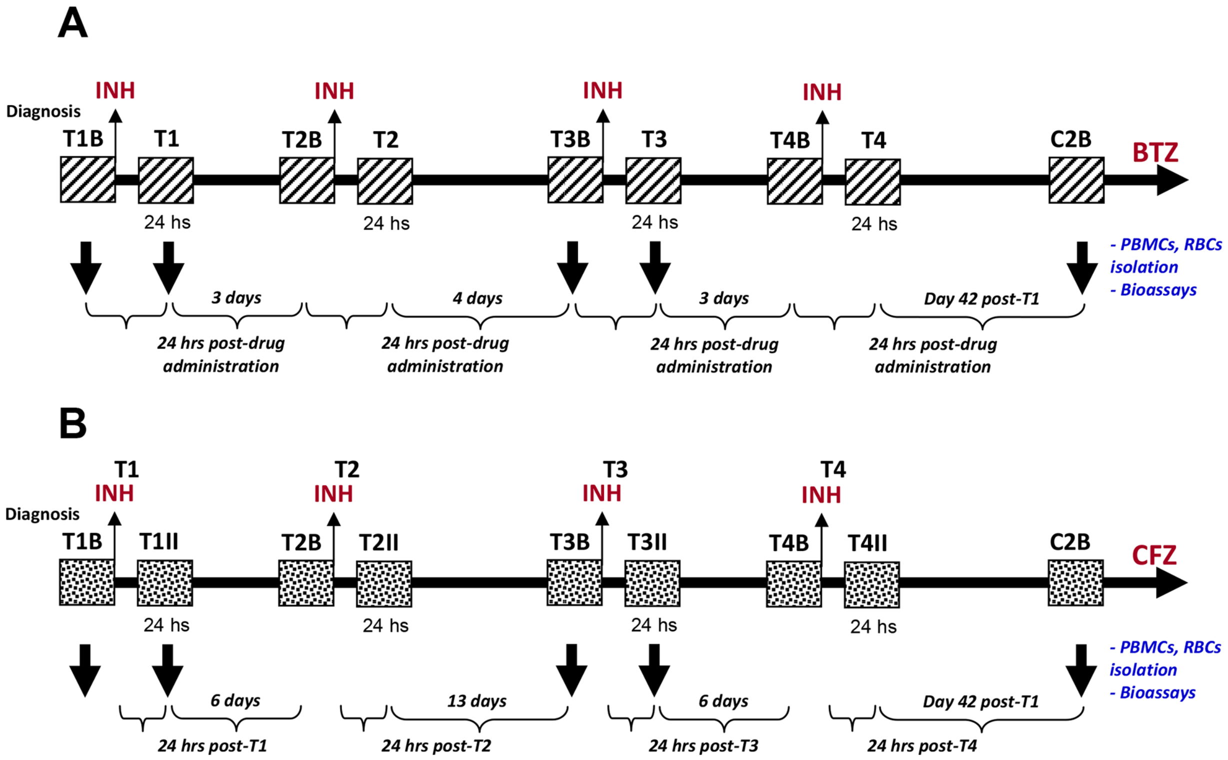

Supplementary Figure 2: Schematic representation of the experimental design and blood sampling time points during therapeutic treatment of MM patients with BTZ **(A)** or CFZ **(B)**. Patients were treated with standard doses of BTZ or CFZ as indicated; arrows denote the five time points (T1B, T1, T3B, T3 and C2B) where blood was collected for PBMCs, RBCs isolation and downstream bioassays. C2; Cycle 2 of treatment.

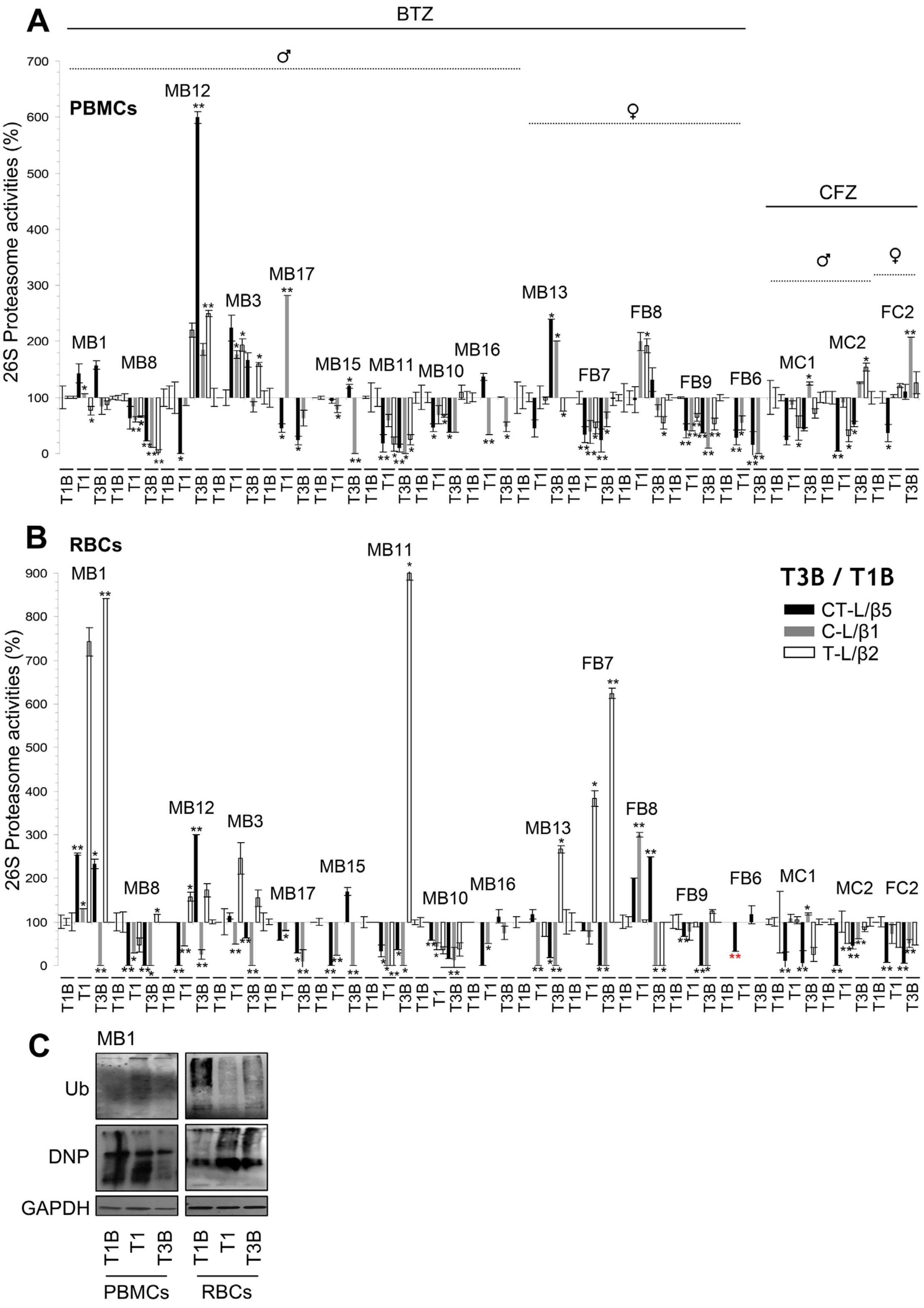

Supplementary Figure 3: Proteasome activities in PBMCs and RBCs isolated from BTZ- or CFZ-treated MM patients (time point T3B). Relative (%) CT-L, C-L and T-L proteasome activities at time points T1 and T3B (*vs*. T1B; see Supplementary Figure 2) in PBMCs **(A)** and RBCs **(B)** isolated from shown MM patients treated with BTZ or CFZ. **(C)** Representative immunoblotting analyses of PBMCs and RBCs samples from patient MB1 at time points T1 and T3B *vs*. T1B. GAPDH probing in (C) was used as reference for total protein input. MB: Male BTZ; FB: Female BTZ; MC: Male CFZ; FC: Female CFZ. Bars, ± SD. $^{*}P < 0.05$; $^{**}P < 0.01$.

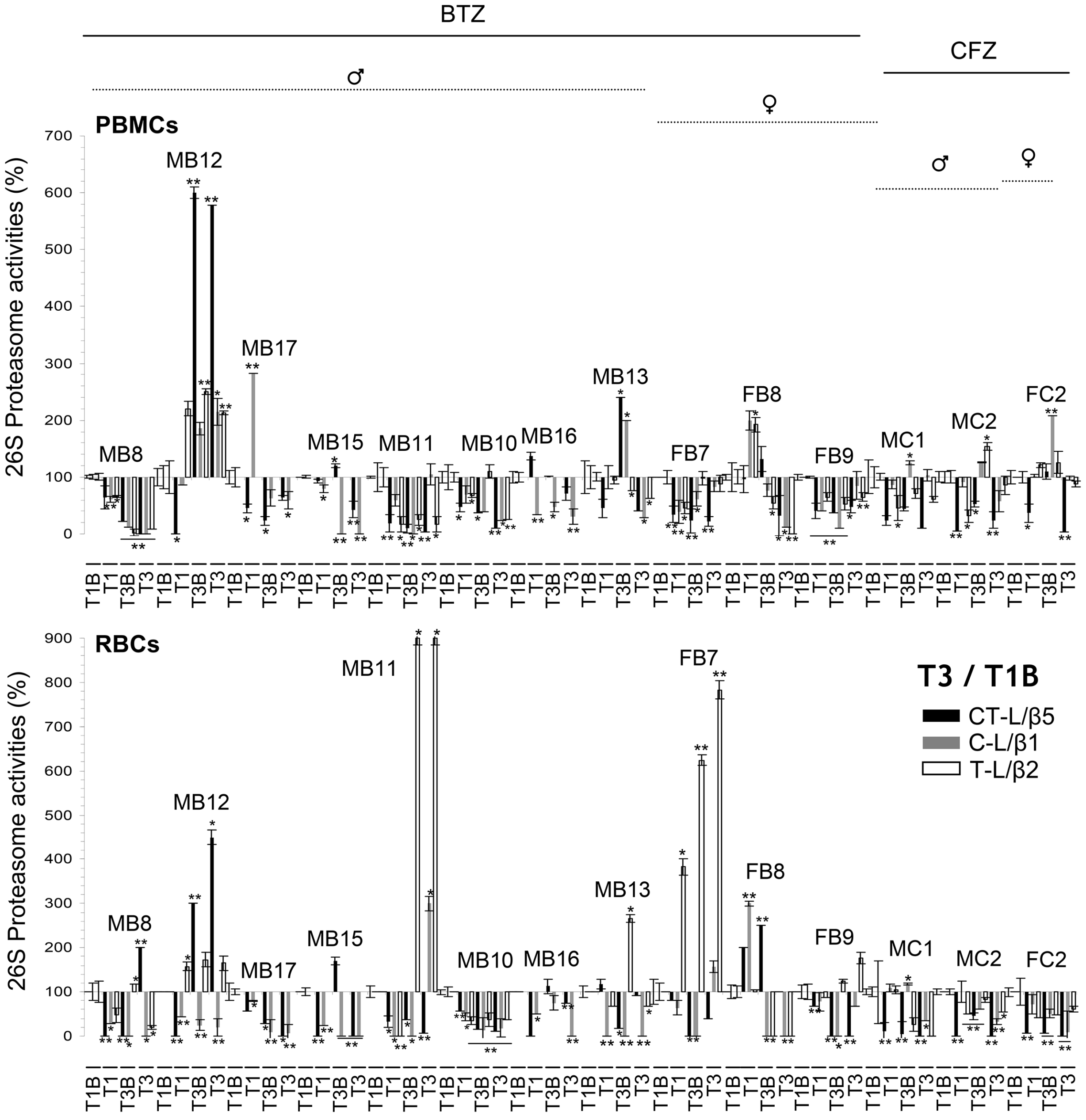

Supplementary Figure 4: Proteasome activities at time point T3 (*vs*. T1B) in PBMCs and RBCs isolated from MM patients treated with BTZ or CFZ. Relative (%) CT-L, C-L and T-L proteasome activities at time points T1, T3B and T3 (*vs*. T1B) in PBMCs **(A)** and RBCs **(B)** isolated from MM patients treated with BTZ or CFZ. Bars, ± SD. *P < 0.05; **P < 0.01.

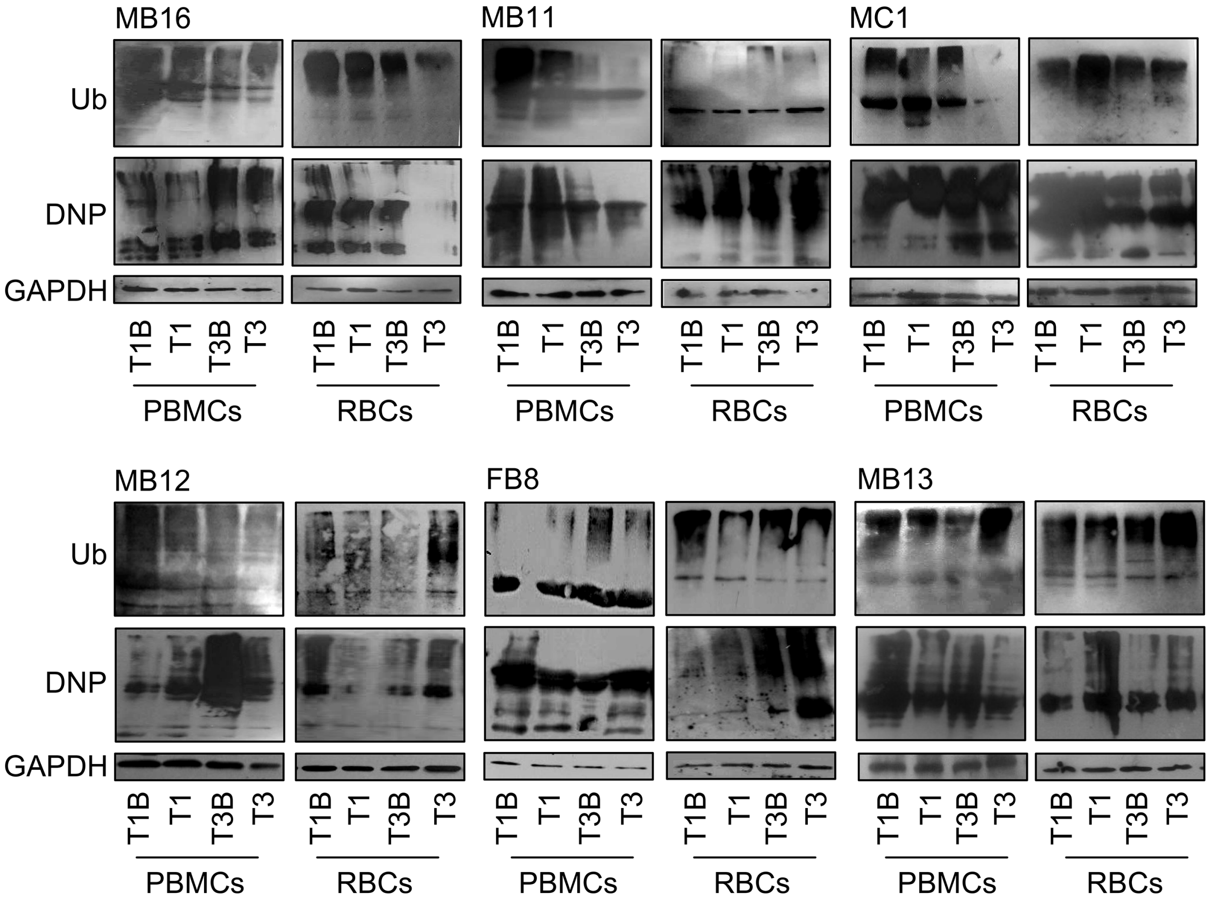

Supplementary Figure 5: Proteome ubiquitination and/or carbonylation in PBMCs and RBCs of MM patients treated with BTZ or CFZ do not correlate with proteasome peptidases activities. Representative immunoblotting analyses of PBMCs and RBCs samples isolated from shown patients at time points T1, T3B and T3 (*vs*. T1B). Blots were probed with anti-Ub (total ubiquitination) and anti-DNP (carbonylation) antibodies. GAPDH probing was used as reference for total protein input.

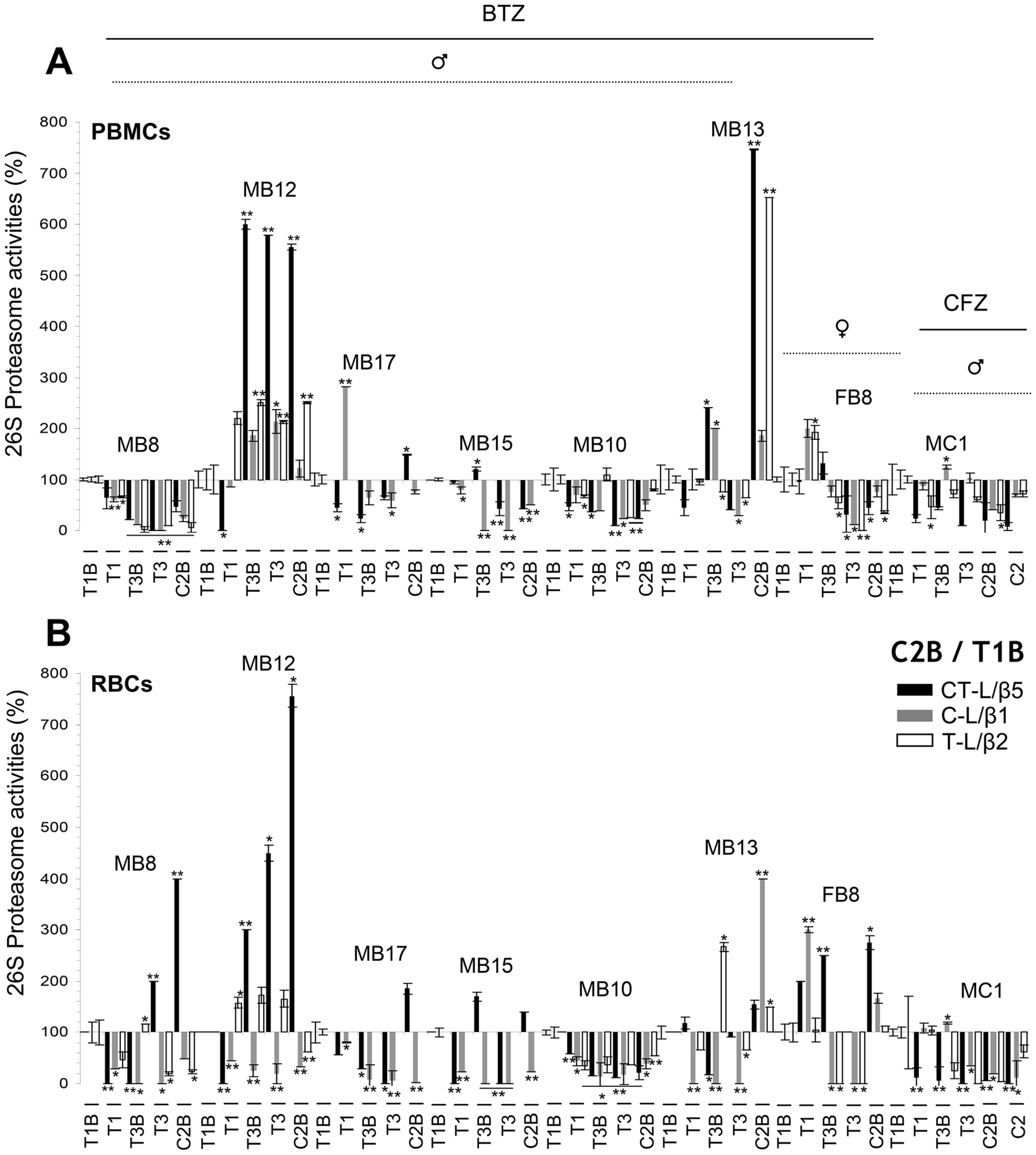

Supplementary Figure 6: Proteasome activities in PBMCs and RBCs isolated at time point C2B from BTZ or CFZ treated MM patients. Relative (%) CT-L, C-L and T-L proteasome activities at time points T1, T3B, T3 and C2B (*vs*. T1B) in PBMCs **(A)** and RBCs **(B)** isolated from MM patients treated with BTZ or CFZ. In patient MC1 we also included time point C2 (24 hrs post C2B). Bars, ± SD. *P < 0.05; **P < 0.01.

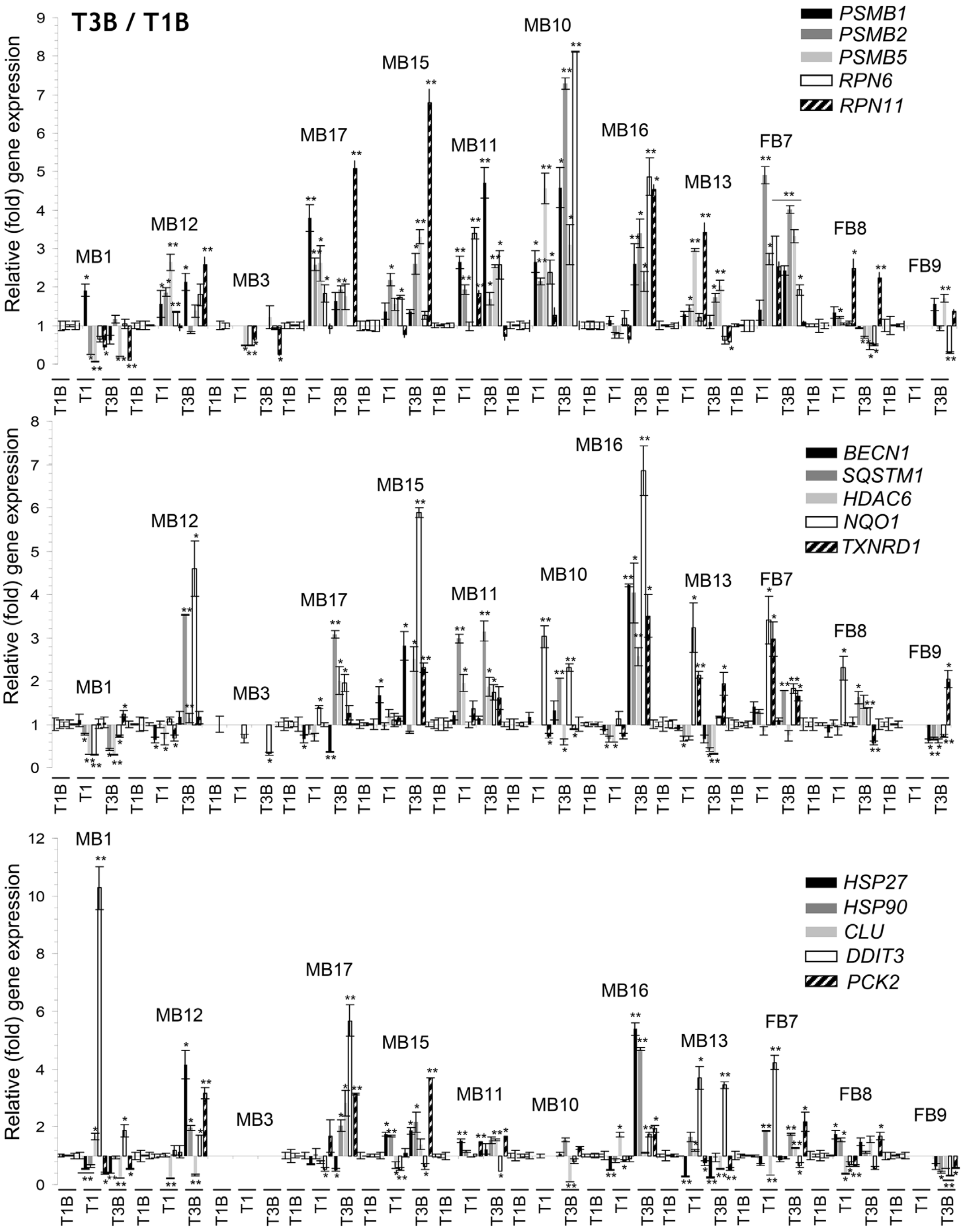

Supplementary Figure 7: Differential genomic responses in PBMCs of MM patients treated with BTZ or CFZ (time point T3B *vs.* T1B). Relative expression levels of the *PSMB1*, *PSMB2*, *PSMB5*, *RPN6*, *RPN11* (proteasome); *BECN1*, *SQSTM1*, *HDAC6* (aggresomes removal/autophagic), *NQO1*, *TXNRD1* (antioxidant), *HSP27*, *HSP90*, *CLU* (chaperone), *DDIT3* (UPR^{ER}) and *PCK2* (metabolic) genes in PBMCs isolated at time point T3B from BTZ or CFZ-treated MM patients. Normalization of gene expression was *vs*. basal values (set to 1) at time point T1B. *GAPDH* gene expression was used as reference for total RNA input. Bars, ± SD. $^{*}P < 0.05$; $^{**}P < 0.01$.

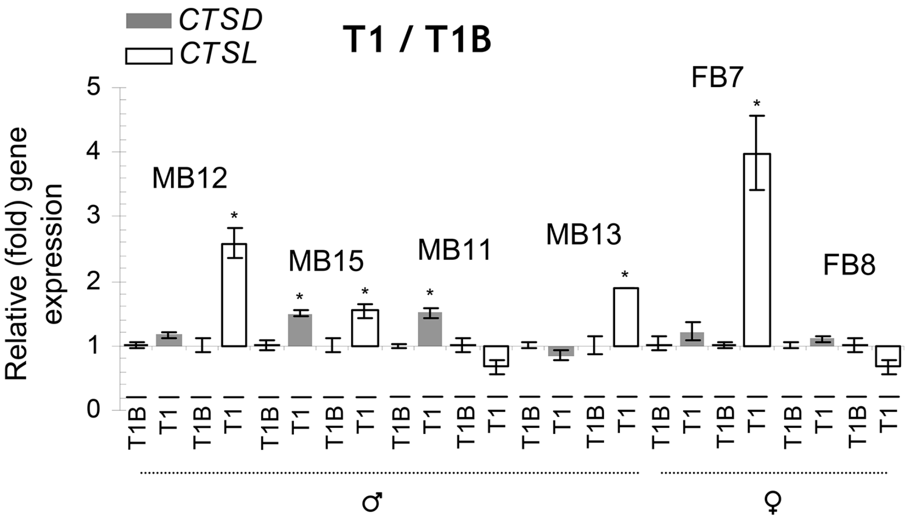

Supplementary Figure 8: Relative expression levels of the *CTSL* and *CTSD* genes in PBMCs isolated from MM patients 24 hrs post-treatment with therapeutic doses of BTZ or CFZ (time point T1; see Supplementary Figure 2). Normalization of gene expression was *vs*. basal values (set to 1) found in samples isolated before treatment initiation (T1B). *GAPDH* gene expression was used as reference for total RNA input. MB: Male BTZ; FB: Female BTZ. Bars, ± SD. $^{*}P < 0.05$.

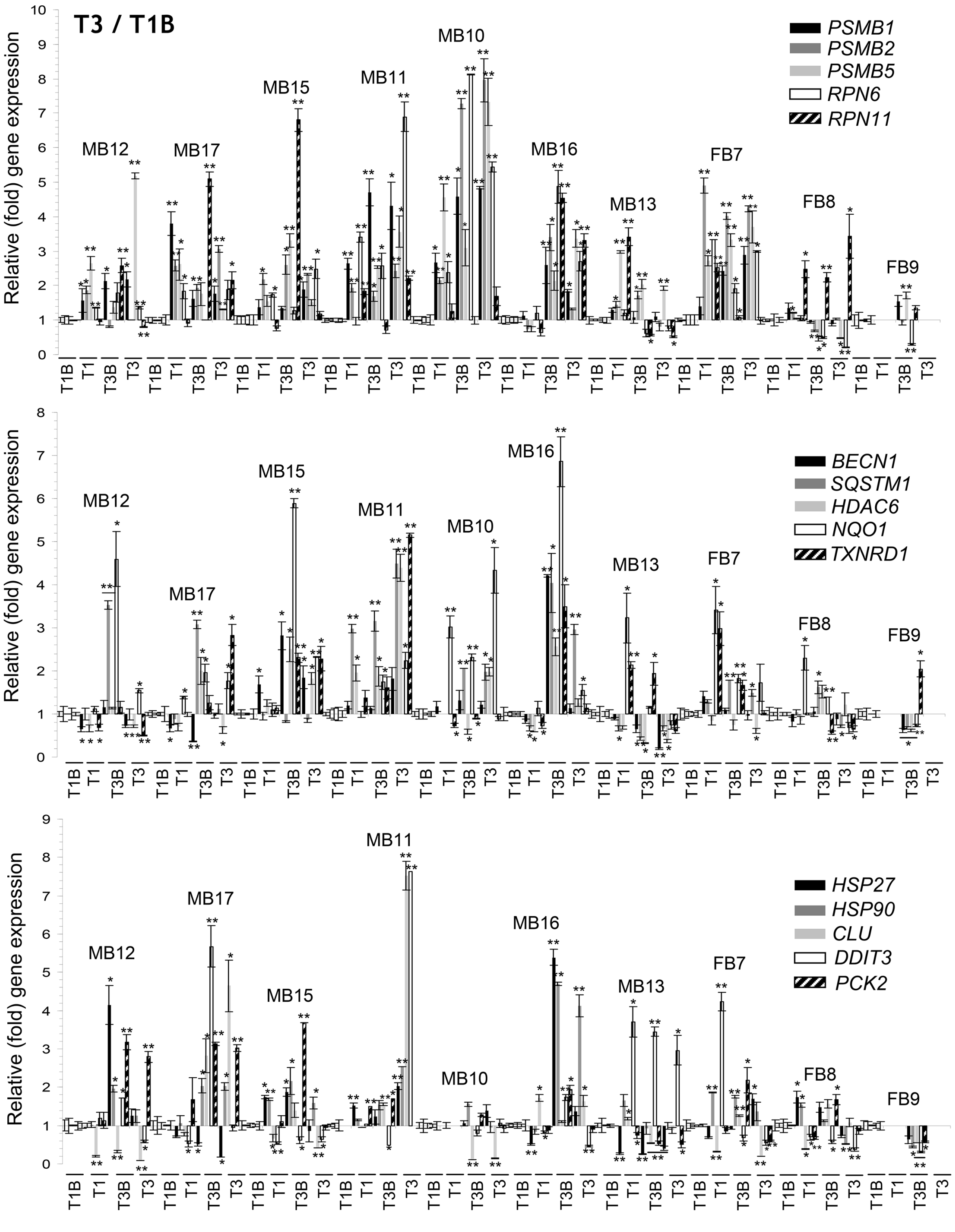

Supplementary Figure 9: Differential genomic responses in PBMCs of MM patients treated with therapeutic PIs (time point T3 *vs*. T1B). Relative expression levels of the shown genes in PBMCs isolated from BTZ or CFZ treated MM patients at time point T3. Normalization of gene expression was *vs*. basal values (set to 1) at time point T1B. *GAPDH* gene expression was used as reference for total RNA input. Bars, ± SD. $^{*}P < 0.05$; $^{**}P < 0.01$.

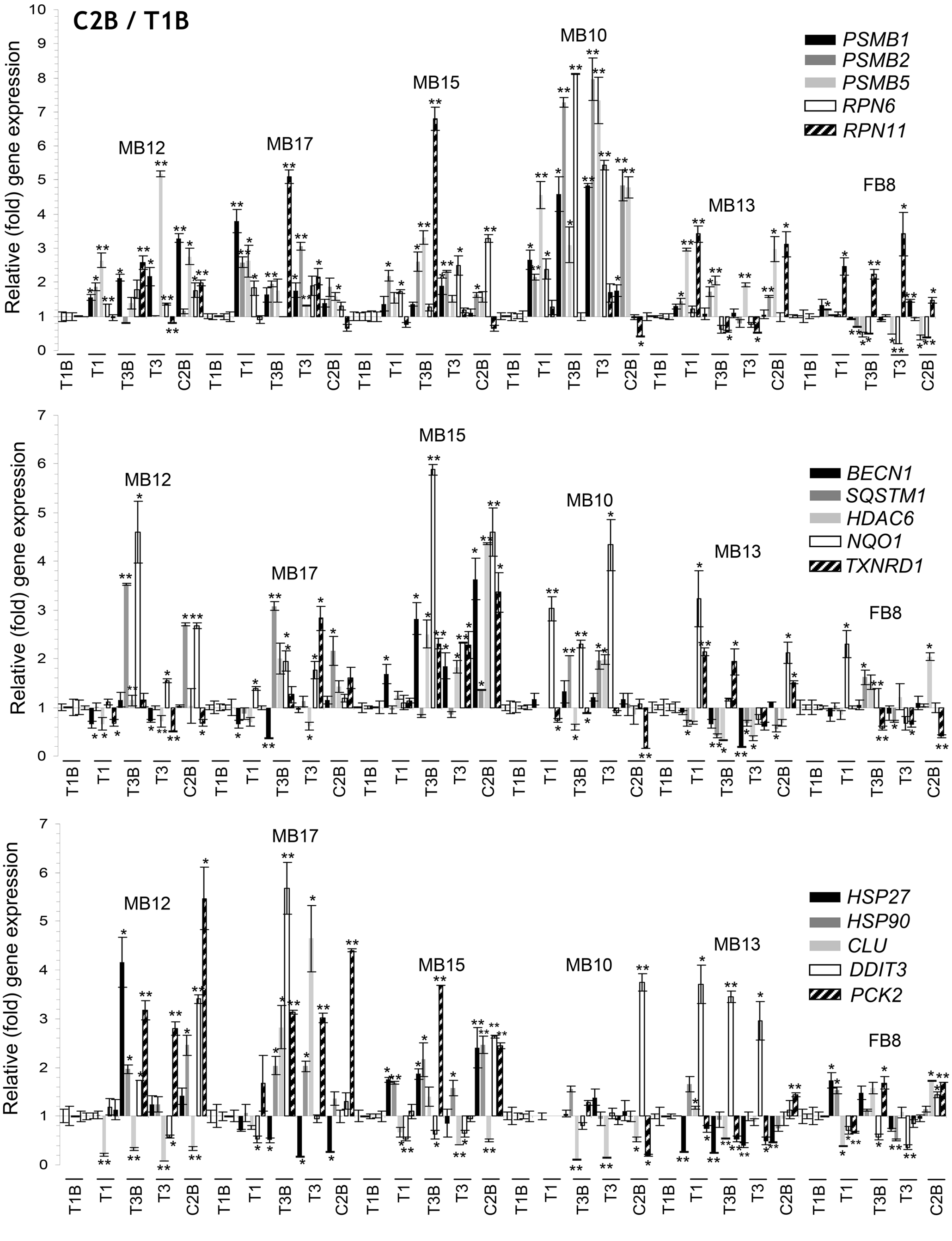

Supplementary Figure 10: Differential genomic responses in PBMCs of MM patients treated with BTZ or CFZ at time point C2B (*vs.* T1B). Relative expression levels of the shown genes in PBMCs isolated from BTZ- or CFZ-treated MM patients at time point C2B; normalization of gene expression was *vs.* basal values (set to 1) at time point T1B. *GAPDH* gene expression was used as reference for RNA input. Bars, ± SD. *P < 0.05; **P < 0.01.

Supplementary Table 1: Pearson's correlations (parametrical).

See Supplementary File 1

Supplementary Table 2: Spearman's correlations (non parametrical).

See Supplementary File 1

Supplementary Table 3: Common correlating parameters (*vs*. IMWG, PFS and OS) in both Pearson's and Spearman's analyses.

See Supplementary File 1
